# Descriptions of Disordered Eating in German Psychiatric Textbooks, 1803–2017

**DOI:** 10.3389/fpsyt.2020.504157

**Published:** 2021-01-14

**Authors:** Lukas Bergner, Hubertus Himmerich, Kenneth C. Kirkby, Holger Steinberg

**Affiliations:** ^1^Archiv für Leipziger Psychiatriegeschichte, Klinik und Poliklinik für Psychiatrie und Psychotherapie, Medizinische Fakultät der Universität Leipzig, Leipzig, Germany; ^2^Department of Psychological Medicine, King's College London, London, United Kingdom; ^3^Department of Psychiatry, University of Tasmania, Hobart, TAS, Australia

**Keywords:** eating disorders, anorexia nervosa, bulimia nervosa, history of psychiatry, German psychiatry

## Abstract

The most common eating disorders (EDs) according to DSM-5 are anorexia nervosa (AN), bulimia nervosa (BN) and binge eating disorder (BED). These disorders have received increasing attention in psychiatry due to rising prevalence and high morbidity and mortality. The diagnostic category “anorexia nervosa,” introduced by Ernest-Charles Lasègue and William Gull in 1873, first appears a century later in a German textbook of psychiatry, authored by Gerd Huber in 1974. However, disordered eating behavior has been described and discussed in German psychiatric textbooks throughout the past 200 years. We reviewed content regarding eating disorder diagnoses but also descriptions of disordered eating behavior in general. As material, we carefully selected eighteen German-language textbooks of psychiatry across the period 1803–2017. Previously, in German psychiatry, disordered eating behaviors were seen as symptoms of depressive disorders, bipolar disorder or schizophrenia, or as manifestations of historical diagnoses no longer used by the majority of psychiatrists such as neurasthenia, hypochondria and hysteria. Interestingly, 19th and early 20th century psychiatrists like Kraepelin, Bumke, Hoff, Bleuler, and Jaspers reported symptom clusters such as food refusal and vomiting under these outdated diagnostic categories, whereas nowadays they are listed as core criteria for specific eating disorder subtypes. A wide range of medical conditions such as endocrinopathies, intestinal or brain lesions were also cited as causes of abnormal food intake and body weight. An additional consideration in the delayed adoption of eating disorder diagnoses in German psychiatry is that people with EDs are commonly treated in the specialty discipline of psychosomatic medicine, introduced in Germany after World War II, rather than in psychiatry. Viewed from today's perspective, the classification of disorders associated with disordered eating is continuously evolving. Major depressive disorder, schizophrenia and physical diseases have been enduringly associated with abnormal eating behavior and are listed as important differential diagnoses of EDs in DSM-5. Moreover, there are overlaps regarding the neurobiological basis and psychological and psychopharmacological therapies applied to all of these disorders.

## Introduction

The diagnosis and treatment of eating disorders (EDs) is an important domain of psychiatry. DSM-5 dedicates a separate chapter to eating disorder diagnoses. Anorexia nervosa (AN), bulimia nervosa (BN) and binge eating disorder (BED) are noted as the clinically most serious and prevalent conditions (1). Additional distinct EDs in DSM-5 include pica, rumination and avoidant-restrictive food intake disorder (ARFID). Prevalence rates of AN and BN are 10 and BED 2 times higher in women than men (1). One in six young women experience an ED (2). Life-time incidence is estimated up to 4% for AN and 2% each for BN and BED (3). Twelve-month prevalence is 0.4% for AN and 1–1.5% for BN. Twelve-month-prevalence of BED in adults is estimated at 0.8–1.6%. Mortality rates for AN at 5 to 20% are the highest of any psychiatric disorder (4) exceeding schizophrenia and depression [(5), p. 426]. Typical causes of death are somatic complications due to food refusal and starvation, but suicide is also common accounting for one in five deaths (4). Although mortality rates in BN are significantly lower than in AN, bulimia is associated with serious complications, such as electrolyte and pH disturbances, and tooth erosion [(5), p. 425]. Recovery rates of 52% in AN and BN in a 6-year follow up (6) indicate the need for more effective treatment.

As well as serious implications for the individual, EDs have significant economic consequences. It is estimated that ~€ 1 trillion per year is spent on the ~20 million patients with eating disorders in the European Union. The direct costs of health care are the same quantum as for anxiety and depression, indirect costs due to burden of disease are even higher (7).

In the English-language literature, the conceptualization of eating disorders as discrete diagnostic entities has developed over the past 150 years. Anorexia nervosa (AN) was first described in 1873 by the French physician Ernest-Charles Lasègue and by the English physician and neurologist William Gull (8). The first description of bulimia nervosa (BN), by Otto Dörr-Zegers, a Chilean of German descent, in the Revista Chilena de Neuro-Psiquiatria (1972), was exceeded in recognition and influence by the description by the British psychiatrist Gerald Russell in Psychological Medicine in 1979 (9, 10). Binge eating disorder (BED) as a separate diagnostic entity debuted as recently as 2013, the time of its inception in DSM-5 (1). The classification of eating disorders is subject to ongoing change. Diagnostic criteria for AN and BN have been subject to frequent alteration. The chapter on eating disorders was extensively revised for DSM-5 and further changes are under consideration for the upcoming edition, including possible new diagnostic entities such as Night Eating Syndrome and Purging Disorder (11, 12).

In the German-language literature, the historical development has been strikingly different. Systematic reviews have already shown that eating disorders received little attention in German-speaking psychiatry until the 20th century. This resulted in a small number of published articles, while in France and England numerous articles on this particular field were published at the same time [(13), p. 204–13, (14)].

Our aim was to investigate whether this low reception also applies to the most common textbooks of that time.

Textbook accounts of disordered eating behavior and related diagnostic entities are of particular value in discerning historical developments. Textbooks generally present a systematic account of the field of psychiatry, incorporate received views of the profession at a given time, detail perspectives promoted by the authors, and indicate formative influences and trends in education and training of psychiatrists and other medical practitioners. This article examines the representation of disordered eating behavior and EDs in German-language textbooks over the past two centuries, in particular addressing the following questions:

- When were eating disorders first mentioned as discrete diagnoses?
- Which other psychiatric diseases were associated with disturbed eating habits?
- What explanations were given for disordered eating behavior?
- In how much detail and to what extent did influential psychiatrists address these issues?
- What continuities and changes can be observed in the conceptualization of disordered eating behavior and diagnostic categories of eating disorders across these more than 200 years?
- Finally, from the perspective of today's clinical practice and available research findings:
- Are there any concepts that are still valid today?
- To what extent do current research findings support or refute the ideas of the past?

## Materials and Methods

In order to gain a comprehensive picture of changing perspectives on disordered eating behavior and eating disorders in the past 200 years of German academic psychiatry, we compiled a list of 18 representative textbooks, taking into account the influence of the authors and distribution of year of publication across the period. This method has been previously applied in a longitudinal historical study (15).

For the selection of literature, we decided on the following strategy: We intentionally chose to focus on psychiatric textbooks because we wanted to present the knowledge that was considered valid at the time, representing the most common and generally accepted views within the clinical and scientific community on disordered eating in the past 200 years. Articles in journals were not taken into consideration, as they did not necessarily represent valid and widespread knowledge.

All the authors considered were professors and therefore their publications had a formative influence on psychiatry and contributed significantly to the teaching of future psychiatrists. Some of the chairs held by the authors of these historic textbooks were even considered to be the most important psychiatric chairs in the world at the time: Heinroth held the world's first psychiatric chair in Leipzig (16). Kraepelin taught in Munich and Heidelberg. He shaped German-speaking psychiatry far beyond his time, introducing clinical-empirical psychiatry and nosology. Other chairs of central importance for psychiatry have been in Vienna (Hoff), Zurich (M. Bleuler) and Breslau (Neumann).

In addition, all considered authors made significant contributions to psychiatry: Griesinger is considered the founder of the scientific and biological phase of psychiatry (17). Eugen Bleuler is considered the founder of the concept of schizophrenia (18). With Kurt Kolle and Helmut Rennert, two of the most important psychiatrists from Eastern and Western Germany are represented as well. Their textbooks have been published in numerous editions, showing their wide and long-term distribution in the field of psychiatry (19, 20). These are exemplary contributions of the cited authors to the field of psychiatry. Further important scientific contributions by the other cited authors can be found in Table 1, listing the cited textbooks and relevant publication data.

**Table 1:** List of authors, lifetime, main contributions to psychiatric science, textbook details.

**Author**	**Life-time**	**Main contributions**	**Textbook title**	**Published place, year**
Johann Christian Reil	1759–1813	Invented the term “Psychiatrie”	Rhapsodieen über die Anwendung der psychischen Kurmethode auf Geisteszerrüttungen	Halle, 1803
Johann Christian August Heinroth	1773–1843	First Chair in psychiatry; founder of psychosomatic medicine	Lehrbuch der Störungen des Seelenlebens oder der Seelenstörungen und ihrer Behandlung	Leipzig, 1818
Wilhelm Griesinger	1817–1868	Founder of scientific psychiatry	Die Pathologie und Therapie der psychischen Krankheiten	Stuttgart, 1845
Heinrich Neumann	1814–1884	Concept of unitary psychosis	Lehrbuch der Psychiatrie	Erlangen, 1859
Richard v. Krafft-Ebing	1840–1902	Sexology	Lehrbuch der Psychiatrie	Stuttgart, 1879
Emil Kraepelin	1856–1926	Empirical psychiatry and nosology	Psychiatrie (8^th^ edition)	Leipzig, 1909–15
Karl Jaspers	1883–1969	Psychopathology	Allgemeine Psychopathologie	Berlin, 1913
Eugen Bleuler	1857–1939	Research in schizophrenia	Lehrbuch der Psychiatrie	Berlin, 1916
August Bostroem	1886–1944	Research in Mb. Wilson, Bostroem paralysis	Kurzgefasstes Lehrbuch der Psychiatrie (4^th^ edition)	Leipzig, 1941
Oswald Bumke	1877–1950	Psychoanalysis, Degeneration theory	Lehrbuch der Geisteskrankheiten (6^th^ edition)	Munich, 1944
Karl Leonhard	1904–1988	Classification of endogenous psychoses	Grundlagen der Psychiatrie	Stuttgart, 1948
Kurt Kolle	1898–1975	Combined psychiatry and neurology	Psychiatrie (4^th^ edition)	Munich, 1955
Hans Hoff	1897–1969	Forensic psychiatry	Lehrbuch der Psychiatrie	Basel, 1956
Gerd Huber	1921–2012	First description of coenesthetic schizophrenia	Psychiatrie	Stuttgart, 1974
Manfred Bleuler	1903–1994	Endocrinologic psychiatry	Lehrbuch der Psychiatrie (15^th^ edition)	Berlin, 1983
Helmut Rennert	1920–1994	Leading psychiatrist in German Democratic Republic	Neurologie und Psychiatrie sowie Grundzüge der Neuropsychiatrie des Kindes- und Jugendalters (8^*th*^ edition)	Leipzig, 1987
Rainer Tölle	1932–2014	Sleep deprivation treatment for depression	Psychiatrie (17^th^ edition)	Berlin, 2014
Frank Schneider	1958	Forensic psychiatry, neurobiology of schizophrenia	Facharztwissen Psychiatrie und Psychotherapie (2^nd^ edition)	Berlin, 2017

Inclusion criteria comprised: textbook of psychiatry in German language; authored by a professor of psychiatry at a German-speaking university; author a recognized authority in psychiatry; published between 1800 and 2017.

**Figure 1:**
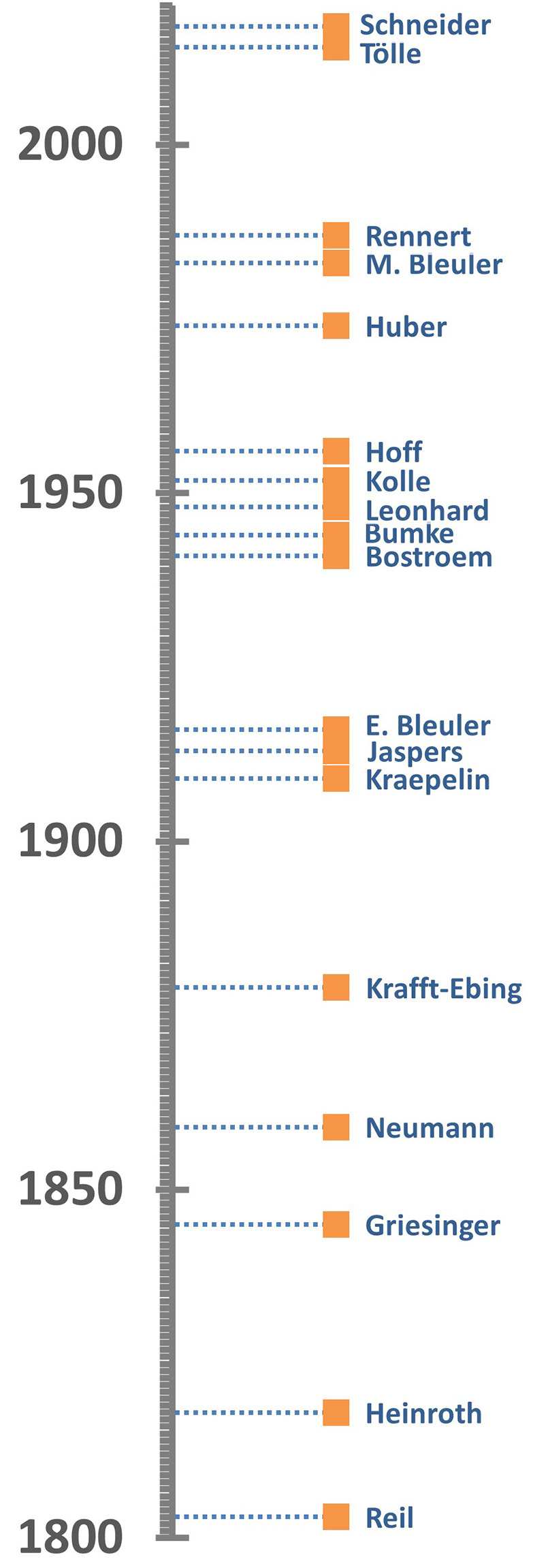
Timeline showing the distribution of the years of publication of the textbooks in this study.

Eighteen textbooks were identified, spanning publication years 1803–2017. The timeline is presented in Figure 1.

In each textbook, relevant passages on eating disorders or their symptoms, such as food denial, emaciation or cravings, were identified, excerpted, and sorted thematically. Historical perspectives and individual views of the authors that shaped their eating disorder narrative were identified. Points of similarity and difference between these historical perspectives and present-day conceptualization of and research findings on eating disorders are summarized and discussed.

## Results

### Eating Disorders as Distinct Diagnostic Entities

The first author to mention eating disorders as a separate disease entity in a German textbook is Gerd Huber in 1974. Huber describes “anorexia mentalis,” characterized by refusal to eat, substantial weight loss, amenorrhea, use of appetite inhibitors and laxatives. This is noted to be most common in pubertal women, Huber discusses delayed “psychosexual maturation” as well as rejection of female roles as possible triggers [(21), p. 270]. This explanation is repeated in later textbooks by Manfred Bleuler and Helmut Rennert (20, 22). Only Rennert mentions fear of obesity as a possible causal factor (20). Tölle and Windgassen emphasize the pathophysiologic role of body image distortion in their current textbook (23).

In our sample, eating disorders as distinct diseases are found in all textbooks published after 1974. However, initially, only AN was listed as an eating disorder. Another 40 years elapsed before BED and BN appeared as independent diagnoses, in the current textbook by Tölle and Windgassen (23). Prior to 1974, disturbed eating behaviors were not categorized as a separate diagnostic condition, even though such behaviors were mentioned as symptoms accompanying a variety of psychiatric disorders.

### Disordered Eating in Depressive Disorders

**Figure 2:**
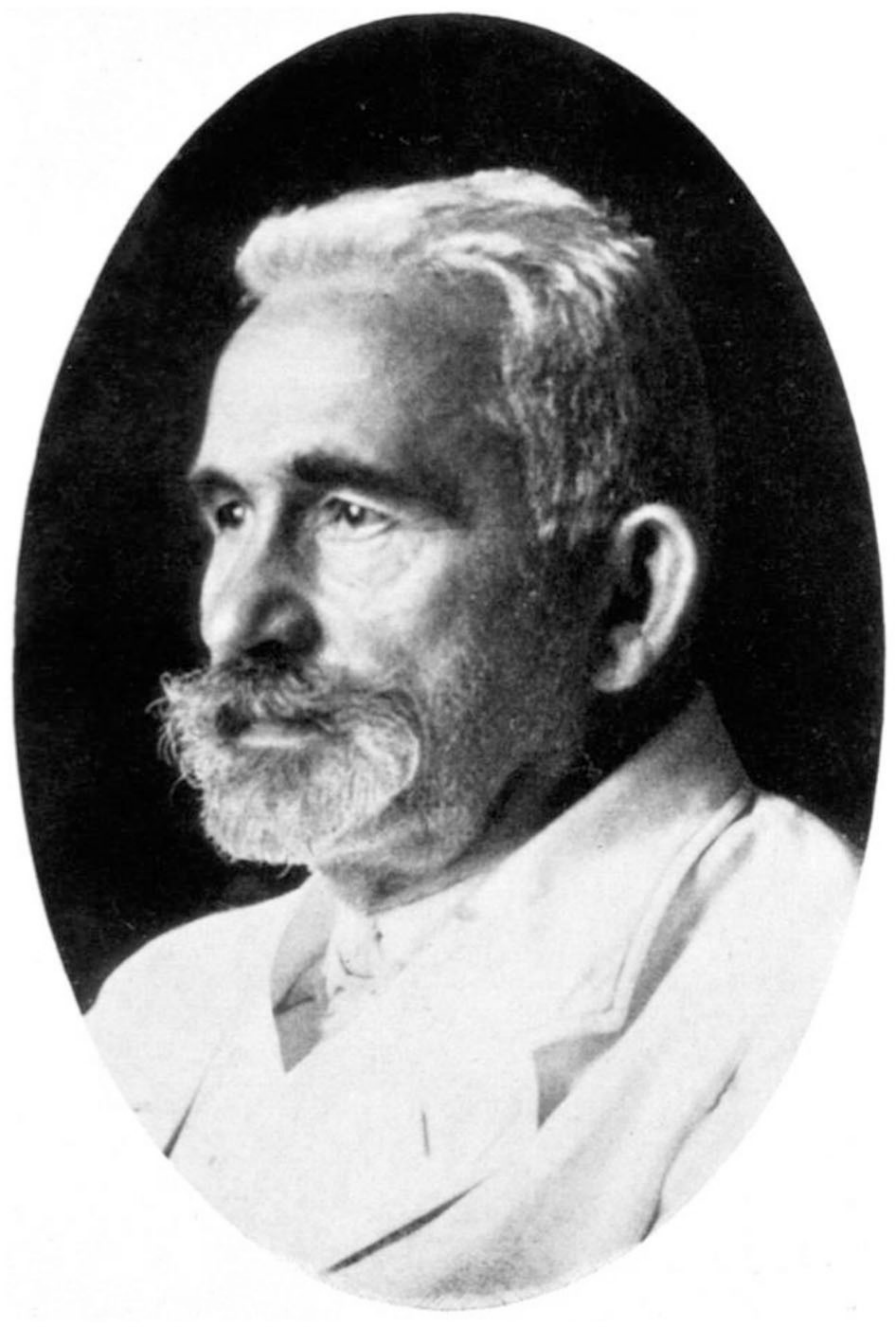
Emil Kraepelin measured body weight attentively in order to comprehend the course and prognosis of mental disorders, at the beginning of 20th century. Source: In public domai, URL: https://wellcomecollection.org/works/mw4ah4kq.

Abnormal eating habits are described extensively as symptoms of depressive disorders. All considered authors mention changes in appetite, food intake or body weight as symptoms of depression. As early as 1803, Johann Christian Reil described loss of weight and emaciation as symptoms of “melancholy” (24). Johann Christian August Heinroth from Leipzig added reduced appetite a few years later (25). Over the next 200 years, loss of weight and appetite are consistently mentioned as common features of depression (5, 21–35). A number of explanations of loss of appetite in depression are discussed. Several authors mention delusions of poverty, leading to patients fearing they will die of starvation (20, 24, 28, 29). Reil describes a patient who believed that voluntarily starving oneself to death was preferable to succumbing to the starvation that inevitably and inexorably accompanied (imagined) dire poverty. As a potential therapeutic approach, Reil suggested reassuring the patient the food provided was free of charge (24). Loss of appetite and weight were linked to feelings of guilt. Wilhelm Griesinger and Krafft-Ebing, the former acknowledged as a pioneer of scientific psychiatry, described fasting as a strategy of sinners to atone for their sins (26, 28). Religious motives are also mentioned by Emil Kraepelin (Figure 2) and persist in Rennert's 1987 textbook where the conviction of having sinned is seen as a reason for loss of appetite, along the lines of a penitential fast [(20), p. 278, (29)]. Finally, several psychiatrists mention delusions of worthlessness (*Mikromanie*) as a reason for reduced food intake. Affected patients believe they don't deserve food (27, 29, 31, 35). Krafft-Ebing describes this, in extreme form, as based on nihilistic delusions. Affected patients deny their very existence, negating the need for food [(28), vol. 2, p. 28]. Thus, delusions are presented as one definitive explanation for food refusal in depressive patients.

Krafft-Ebing formulated a remarkable approach to monitoring the course of depressive illness, regularly measuring body weight for early detection of depressive episodes. In 1879 he states that a masked depression (*larvierte Depression*) may initially present somatic symptoms, even before characteristic symptoms of depressed mood occur. Thus, “those swallowed tears, those inner wounds that have been covered by smiles, pride and lies for an extremely long time” can lead to emaciation, digestive problems, amenorrhea or irregular menstruation before mood alteration occurs [(28), vol. 1, p. 129]. Such physical findings are also referred to in Frank Schneider's current textbook as typical somatic complications of AN [(5), p. 424]. However, food intake does not play a key role in Krafft-Ebing's account of masked depression, so it does not appear to constitute an early depiction of AN.

### Food Refusal as a Suicidal Act

Beginning with Heinroth, food refusal as a suicidal act was a focus of attention in the 19th and early 20th century (25–30). Heinrich Neumann from Breslau, founder of the concept of unitary psychosis, held that the danger of suicide persisted even after beginning force feeding, since the patient could fall back on suicide methods other than food refusal [(27), p. 207]. Krafft-Ebing considered food refusal a rare form of suicidal act [(28), vol. 2, p. 28]. Kraepelin names suicide as one of the most frequent motives for the refusal to eat [(29), vol. 1, p. 618]. Kraepelin is the only author to mention suicide by eating inedible objects such as “nails, stones, fragments of glass, animals,” providing the patient could consciously overcome feelings of disgust [(29), vol. 1, p. 364]. Suicide by starvation associated with depression is not mentioned in the later textbooks. It was not until 1987, that Rennert claimed that food refusal may be an accompaniment of suicidal intent in patients with anorexia [(20), p. 457]. Finally, Tölle and Windgassen explicitly mention eating disorders in their chapter on suicidality where they refer to anorexia nervosa as a form of “chronic suicide.” Obesity is also noted as a possible variant of this suicide type [(23), p. 100, 126].

### Disordered Eating Behavior in Schizophrenia

Altered eating behavior is also found in descriptions of schizophrenia, mostly in relation to reduced food intake. Delusions and hallucinations are implicated as valid explanations. In 1818, Heinroth states the preoccupation with intense delusions in “madness (paranoia)” may lead to neglect of sleep and eating. Furthermore, abeyance of appetite can occur as a result of incessant preoccupation with supernatural phenomena and religious writings [(25), vol. 1, p. 296–304].

A number of authors link the content of delusions to possible effects on eating (15, 17, 18, 21–24, 28). Griesinger describes delusions of poisoning and that human flesh had been offered to eat, as leading to food refusal [(26), p. 77–83]. Neumann considered delusions to be the most common reason for food refusal. In addition, he describes strategies to persuade affected patients to eat. The treating physician might partake of the food on offer to convince the patient it is safe. One patient had even devised his own strategy to circumvent starvation, solely eating eggs, since these could not contain human flesh [(27), p. 206]. Krafft-Ebing describes delusions that one's own body does not exist or has already died, and delusions of poisoning associated with paranoia as leading to food refusal. He also notes that certain delusions could lead to increased appetite. Affected patients were convinced of “having several children in their womb, or of being double bodied” [(28), vol. 1, p. 65f., 103]. Reil had previously described a patient with a delusional belief of double existence, who ate double the normal serves of food [(24), p. 80]. Overall, however, delusions causing reduction in food intake dominate in the literature considered. Describing “dementia praecox” (DP), Kraepelin mentions the example of a delusion of state-organized poisoning. Under the “paranoid form” of DP he mentions that a delusion of poisoning may result in only certain foods being eaten or food intake ceased, worsening the overall disease prognosis [(29), vol. 3, p. 696, 842, 896]. In 1941, August Bostroem emphasized food refusal not only as a complication, but also an indication for in-patient treatment in patients with schizophrenia [(32), p. 194]. Gerd Huber describes various vegetative symptoms that may occur in the course of schizophrenia: “Loss of appetite, nausea and vomiting, constipation and diarrhea.” Food intake may be increased or decreased and sudden changes in eating habits may occur [(21), p. 165f.]. In the second half of the 20th century, Huber and Rennert describe delusions of poisoning as a symptom of schizophrenia [(20), p. 278, (21), p. 177]. Tölle and Windgassen also discuss a link between these conditions [(23), p. 198]. Thus, delusions of poisoning have been consistently regarded and taught as a symptom of schizophrenia in the past 200 years of German academic psychiatry and often linked to eating behavior.

Gustatory and auditory hallucinations in schizophrenia are also cited as potential triggers of disordered eating (20, 21, 26–28). This primarily involves the perception of unpleasant tastes. For example, Griesinger notes tastes that are “disgusting, metallic, pungent, rotten, earthy.” Gustatory hallucinations involving a pleasant taste were much rarer [(26), p. 83f.]. In the mid-19th century, Griesinger, Neumann and Krafft-Ebing discussed gustatory hallucinations as leading to delusions of poisoning [(26), p. 83, (27), p. 115, (28), vol. 1, p. 66, 104], only for mention of this link to fall into abeyance for the next 100 years until Huber and Rennert discussed gustatory hallucinations triggering refusal to eat [(20), p. 278, (21), p. 177].

Neumann also described auditory hallucinations as the basis of a case of food denial. Voices stated there was human flesh in the food and commanded a food ban [(27), p. 116]. A few years later, Krafft-Ebing described voices prohibiting food on religious grounds [(28), vol. 2, p. 28].

Abnormal eating habits are also found in the descriptions of catatonic and hebephrenic forms of schizophrenia by the great nosologist Kraepelin. In hebephrenic schizophrenia (*läppische Verblödung*), food intake may fluctuate considerably, corresponding to phases of increased and decreased appetite. In megalomania, patients may deny needing food, insisting they are fueled by “supernatural power.” In catatonia, delusions of poisoning are occasionally found [(29), vol. 3, p. 768–76, 810]. Some patients would consume inedible substances, even their own excretions. Patients may rigorously enforce refusal to eat by clenching their teeth, protesting they are neither hungry nor require food [(29), vol. 3, p. 816–29]. Three other leading German psychiatrists, Eugen Bleuler, Karl Leonhard and Manfred Bleuler, describe food denial in patients with catatonia [(22), p. 198, (31), p. 106, (34), p. 121]. Kurt Kolle mentions “pubertal anorexia” (“*Pubertätsmagersucht*”) as a special form of schizophrenia in his textbook from 1955. Describing emaciation and food deprivation as its core symptoms, he discusses parallels to hebephrenic schizophrenia and a possible common pathophysiology. Eating disorders as a distinct category of mental disorders are not described by Kolle (19).

### Increased Appetite in Mania

Descriptions of bipolar disorder, formerly referred to as “mixed states” or “circular insanity” report less consistent connections to eating behaviors. Many authors describe an increase in appetite during a manic phase (25, 26, 28, 29, 34, 35). Griesinger regarded increased appetite as a symptom of mania, attributing it as a lack of satiety [(26), p. 64, 224]. However, he noted that some manic patients cited their great appetite as justification for their claim not to suffer from any disease. Several authors in the first half of the 20th century described reduction of appetite during a manic phase. According to Eugen Bleuler, patients with mania ate not to appease their hunger, but rather in order to occupy themselves. “Submanic” patients with moderate activity, on the other hand, showed a diminished eating behavior [(31), p. 359]. In 1941, Oswald Bumke described a lack of appetite during mania, leading to weight loss [(33), p. 279–88]. The authors of recent textbooks again see mania linked with increased appetite. According to Leonhard, internationally known for his differentiated classification of endogenous psychoses, this could lead to a more vital appearance of the patient [(34), p. 90]. In 1987, Rennert even describes “greed” as a symptom in manic patients [(20), p. 278], however, weight gain is not described as a typical consequence of the increased appetite. Most psychiatrists rather considered weight loss a sign of mania (26, 28, 31, 33–35). A possible explanation for this apparent contradiction can be found in Hans Hoff's textbook, describing increased metabolism in manic patients [(35), p. 403]. Kraepelin alone offers the more nuanced description of increased weight in milder cases of mania, while more severe cases are associated with weight loss [(29), vol. 3, p. 1228].

### Disordered Eating Behavior in Descriptions of Historical Disease Categories

In addition to these psychiatric diagnoses that are well-known today, abnormal eating habits have also been described in diseases that are no longer diagnosed in the present day, have been dissolved into other concepts or bear another name.

Kraepelin describes “neurasthenia” as a disease state, caused by overload of the human body and mind, resulting in exhaustion and loss of performance. Another symptom is lack of appetite; prolonged refusal to eat may also cause stomach discomfort, which can be counteracted by eating small amounts of food frequently [(29), vol. 4, p. 1401, 1465]. According to Oswald Bumke, malnutrition is not only a symptom but may cause neurasthenia. He also mentions stomach discomfort as a possible symptom in addition to other vegetative disorders in the cardiovascular system and gastrointestinal tract, with decreased appetite being the generic consequence [(33), p. 297]. Hans Hoff departs from this description of neurasthenia. He too describes manifold symptoms of the digestive tract, such as stomach pain or gastroesophageal reflux. However, with respect to eating behaviors, Hoff describes as typical a “ravenous appetite” at night resulting in overweight. Rumination of already eaten food may also occur [(35), p. 589]. The last textbook in our series with an entry for “neurasthenia” was published in 1974 by Gerd Huber. Therein he states that both reduced and increased appetite can be observed in this disease, manifesting as “loss of appetite” or “bulimia and polyphagia.” He brackets neurasthenia and *anorexia mentalis* as typical representatives of psychiatric disorders showing both physical and psychiatric symptoms [(21), p. 258–64].

In the 19th and early 20th century, occasional descriptions of abnormal food intake are found in “hypochondria.” Griesinger described heart problems, headaches and sleep disturbances as well as indigestion and lack of appetite as possible symptoms of this condition. Weight loss only occurred in cases of concurrent physical illness [(26), p.159 f.]. According to Neumann, on the other hand, patients with hypochondria focus on meticulously choosing the foods eaten [(27), p. 95, 160]. Krafft-Ebing describes a preference for inedible food. Patients consumed “spiders, toads, worms, human blood,” as they hoped for a healing effect [(28), vol. 1, p. 66]. Furthermore, hypochondriacal symptoms could also occur in other psychiatric disorders. Krafft-Ebing describes “hypochondriacal melancholy” as an example: Affected patients claimed that their intestines were blocked and therefore they could not eat anything [(28), vol. 2, p. 28]. Eugen Bleuler describes a case of “schizophrenic hypochondria”: one patient was convinced that she was suffering from an ileus and that the food in the intestine was going to rot. Subsequent intake of laxatives led to weight loss [(31), p. 320]. More generally, Karl Jaspers, well-known today for his seminal work on descriptive psychopathology, mentions hypochondriacal symptoms as a complication of psychiatric illnesses that could cause severe weight loss [(30), p. 129].

Descriptions of abnormal eating habits are also found in accounts of “hysteria.” Although Kraepelin viewed hysteria as an inconsistently described disease, he regarded instability of affect as its cardinal symptom, which could cause both mental and somatic sequelae. Affects could perturb body functions: Thus, “nausea, choking movements, and vomiting” would be an expression of disgust, loss of appetite a sign of sadness [(29), vol. 4, p. 1548–56]. Ingestion of food may be blocked by spasm of the esophagus, and vomiting was a frequently observed symptom. Nutritional status might only be slightly impaired, but in some cases could be greatly reduced. Further, the amount of food eaten by patients could be reduced over months or years so extensively that it was barely sufficient for survival. Young women most commonly suffered from this disease, with amenorrhea a typical complication. To treat the above symptoms, Kraepelin recommended a “mast cure” (a fattening diet regime) [(29), vol. 4, p. 1598–603, 1697]. Eugen Bleuler also saw vomiting, refusal of food and difficulty swallowing as symptoms of “hysteria.” However, metabolism would be down-regulated in affected patients so that the weight remained constant [(31), p. 380–7]. Jaspers was concerned with the causes of vomiting, which he too regarded as a symptom of “hysteria.” A stressful feeling that occurs during ingestion would be suppressed and could manifest later through months of vomiting [(30), p. 176]. Bumke's explanation for food refusal was that whilst a self-injurious behavior it served primarily to attract attention. On the other hand, when alone, the patient would eat secretly and deliberately, intentionally deceiving those around them [(33), p. 212f.]. Hans Hoff describes “hysterical anorexia” as a special form of “hysteria” with some parallels to AN. It typically occurred following puberty in young women, who were averse to maturing to womanhood, denying food in order to delay the development of their body. This process would happen unconsciously and might lead to an “endogenous anorexia” [(35), p. 618].

### Somatic Conditions and Altered Brain Function

In addition to psychiatric disorders, somatic conditions are discussed as possible triggers of altered eating behavior, especially diseases of the gastrointestinal tract, endocrinological disorders, and lesions of the brain and pituitary gland.

Pathologies of the gastrointestinal tract were particularly seen as responsible for changes in eating behavior in the 19th century. According to Reil, increased appetite may occur due to increased activity of the nervous system in the gastrointestinal tract [(24), p. 258]. By contrast, Krafft-Ebing considered hypersensitivity of the gastric nerves to cause decreased appetite, leading to a premature feeling of satiety [(28), vol. 1, p. 66]. He also cited inflammatory changes in the gastrointestinal tract, which were also discussed by Kraepelin and Griesinger as a plausible cause of loss of appetite [(26), p. 327, (28), vol. 1, p. 204, (29), vol. 1, p. 618]. In addition, pain could trigger refusal to eat. According to Griesinger, pain sensations could impair food intake and lead to weight loss [(26), p. 29]. Neumann emphasized that abdominal pain in particular could have a significant impact on a person's nutritional status, although gastrointestinal diseases could also lead to increased appetite [(27), p. 59, 79].

In the 20th century, more attention was devoted to the brain than to the gastrointestinal tract. In particular, brain lesions were cited as a somatic cause of altered eating habits. Both increase and decrease in appetite were considered possible. According to Bumke, emaciation may occur as a result of degeneration of brain areas associated with vegetative functions [(33), p. 387]. More generally, Huber stated that a disturbed appetite may result from local brain damage [(21), p. 49]. According to Bostroem, an increased appetite could occur in the course of a frontal-brain lesion and may cause a craving for food [(32), p. 23]. Hoff described a lesion of the diencephalon as a possible trigger for periodic food cravings [(35), p. 195]. According to Leonhard, increased intracranial pressure could increase or decrease appetite [(34), p. 228].

In addition, panhypopituitarism (“Morbus Simmonds”), a hypofunction of the hypophysis, was previously considered responsible for emaciation. In the 1940's however, Bostroem and Leonhard described personality changes, altered affectivity and cognition as symptoms of the disease, but did not mention possible effects on body weight [(32), p. 60, (34), p. 140]. Not until 1974 did Huber cite pituitary disease in the differential diagnosis of “anorexia mentalis” [(21), p. 271]. Even Manfred Bleuler did not equate these two clinical pictures. Rather, he emphasized that AN is psychogenic in origin and not an endocrinopathy. Pituitary hypofunction may be considered as a differential diagnosis [(22), p. 357]. Rennert also considered “Simmond's cachexia” in 1987 as a differential diagnosis of anorexia [(20), p. 278]. In the current textbook by Frank Schneider, panhypopituitarism only receives historical attention along the lines that at the beginning of the 20th century, anorexia was attributed to malfunction of the pituitary gland according to a publication by the Hamburg pathologist Morris Simmonds. Diseased patients were treated with hormonal therapies or even pituitary transplants, whereas psychotherapy was not used [(5), p. 422].

### Appetite and Body Weight as a Prognostic Factor

In the first half of the 19th century, Heinroth and Neumann regarded body weight and appetite as prognostic markers: they describe increasing appetite and weight as a sign of convalescence in patients with “melancholy” [(25), vol. 1, p. 335]. According to Neumann, the body weight may even exceed the original weight before the disease [(27), p. 189]. Krafft-Ebing, Kraepelin and Jaspers extended the prognostic significance of body weight to all psychiatric illnesses. In the presence of improved psychopathological state, weight gain would be a sign of healing. In the views of Krafft-Ebing and Kraepelin, weight has such prominence that they doubt that successful cure of a mental illness without increase in bodyweight is possible [(28), vol. 1, p. 214f, (29), vol. 1, p. 445, (30), p. 327]. The most unfavorable constellation is considered to be increasing body weight with unimproved psychopathological state, indicating the persistence of the mental disorder [(28), vol. 1, p. 215, (29), vol. 2, p. 892, (30), p. 130].

**Figure 3:**
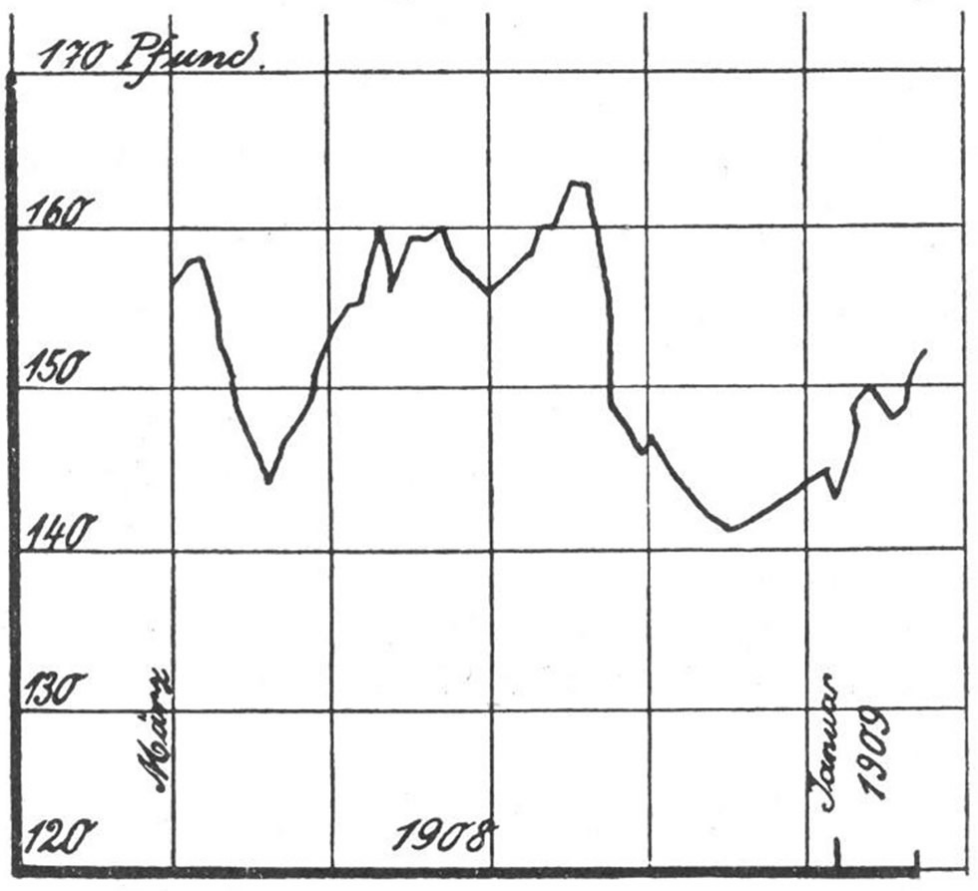
Weight fluctuations in periodic insanity, as reported by Kraepelin. Abscissa: time [~ 1 year]; Ordinate: bodyweight [pounds]. The first drop in weight occurred during a manic phase, with weight regained in convalescence, followed by significant weight loss during a more prolonged major depressive episode. Source: Obtained from [(29), vol. 3, p. 1231].

**Figure 4:**
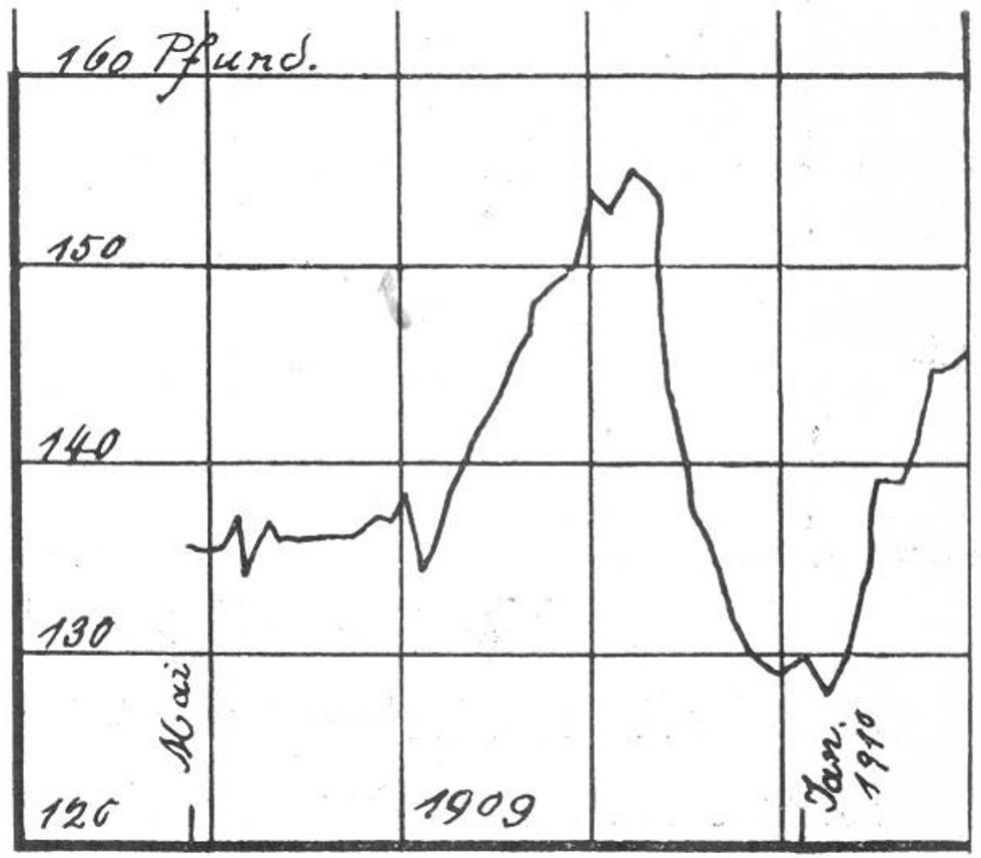
Abscissa: time [~ 1 year]; Ordinate: bodyweight [pounds]. According to Kraepelin, changes in weight indicate the severity of psychiatric symptoms: Whilst the first two slight weight drops occur in mild depressive phases, the following rapid and substantial weight loss represents a severe depressive episode. Source: Obtained from [(29), vol. 3, p. 1231].

Kraepelin reported from his clinical research that body weight could reflect the course and severity of psychiatric disorders. In his four-volume textbook there are illustrations of weight curves with descriptions of the associated course of the disease. Figure 3 illustrates this with regard to a case of “periodic insanity,” an historic diagnosis that resembles today's concept of bipolar disorder. The graph illustrates fluctuations in weight during the course of the disease. The first weight loss represents a manic phase followed by increase in weight indicating a remission. The second drop, on the other hand, represents a depressive phase. The severity of a disease can be measured by the amount of weight fluctuation. Figure 4 describes similar observations in a recurrent depressive disorder with minor and major episodes of symptoms.

In the time following Kraepelin's interest in body weight, the tenet that weight can be a marker for severity and prognosis largely disappears from view. Bumke strongly states the contrary case, that in patients with schizophrenia, weight fluctuations are completely independent of psychiatric symptoms [(33), p. 572]. In later textbooks, weight is no longer mentioned as a prognostic factor in mental disorders.

## Discussion

The review of textbook entries confirms that diet and especially altered eating habits have been addressed in German psychiatric textbooks throughout the past 200 years. The main findings are summarized in Table 2.

**Table 2:** Summary of main findings of review of eating disturbance and disorder content in textbooks.

First mention of EDs	Anorexia nervosa 1974 by Gerd Huber Bulimia nervosa 2014 by Rainer Tölle Binge eating disorder 2017 by Frank Schneider
Disordered eating symptoms described	Food refusal Food craving Weight gain Weight loss Vomiting Eating inedible things
Disordered eating behaviors as symptoms of mental disorders	Schizophrenia Depressive disorders Bipolar disorder Hysteria Neurasthenia Hypochondria
Pathophysiological explanations given for disordered eating	Psychological processes: Guilt, sin, suicidal thoughts and tendencies, attracting attention Psychopathology: Delusions of poisoning, delusions of poverty, nihilistic delusions, gustatory and auditory hallucinations Diseases in GI tract: indigestion, inflammation Dysfunction in certain brain areas: Frontal lobe, diencephalon, pituitary gland

**Figure 5:**
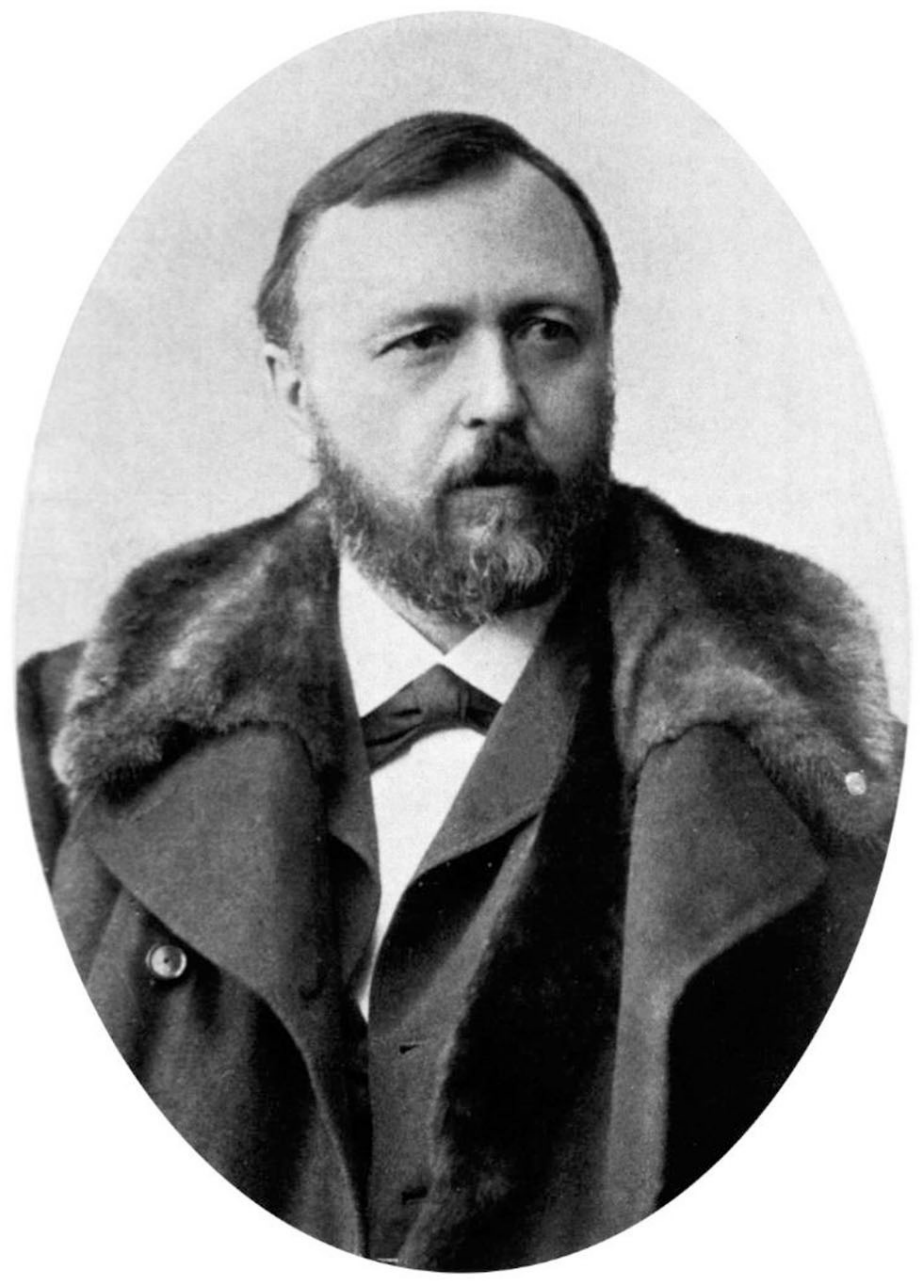
Richard von Krafft-Ebing addressed disordered eating extensively, dedicating a distinct chapter to this topic in his textbook of 1879. Source: In public domain, URL: http://resource.nlm.nih.gov/101420138.

As early as 1879 Krafft-Ebing dedicated a separate chapter in his textbook to disordered eating (Figure 5). In particular, he regarded the nutritional status and appetite as markers for mental health and the prognosis of psychiatric disorders. Changed eating habits, however, were not regarded as an independent disease entity, but rather as symptoms of other mental or somatic diseases. This view has been maintained for almost a century. Only in 1974 Gerd Huber mentions “Anorexia mentalis” as a specific disorder in his textbook [(21), p. 270f.].

In German-speaking psychiatry, eating disorders were mentioned occasionally, as an article by Anton Stichl from 1892 shows: His article on the “Anorexia mentale,” describes the fear of being fat as a causal factor and named self-induced vomiting and lack of insight as characteristics of the disease. This description of the AN is still valid today and let Stichl's publication appear very modern (1, 36). Overall, however, only a few German-language articles featured eating disorders well into the 20th century (14). Our review shows clearly that this also applies to the published textbooks of that time.

This is in contrast to the developments in other European countries, especially France and England: There, Gull and Lasègue described “anorexia hysterica” and “anorexia nervosa” almost simultaneously in 1873 and 1874. Their descriptions resemble today's concepts of AN in many respects, emphasizing the psychogenesis of this disease and rejecting somatic explanations like a disease of the gastrointestinal tract. They also described the therapy as difficult due to a lack of insight and mentioned typical complications such as constipation and amenorrhea (37, 38). The publications aroused great interest in France and England, and numerous articles on this “new disease” were published in specialist journals [(13), p. 204–13].

The late reception in German speaking psychiatry however is particularly noteworthy due to its leading contributions in fields like descriptive psychopathology, diagnosis and neurosciences.

In the following we want to explain reasons for this particular development in the German-speaking world.

### The Role of Somatic and Psychosomatic Medicine in German-Speaking Countries

There is no single universal explanation for the sparse and late reception of eating disorders in the German-language literature. Rather, multiple factors and historical circumstances must be taken in account. An important factor is that eating disorders were not considered the preserve of psychiatrists in German-speaking countries, a review of AN treatment at the end of the 19th century indicated that pediatricians, gynecologists and in particular internists dedicated themselves to those diseases (14). It is likely the multiple physical complications of AN aroused the interest of somatic doctors, thus DSM-5 lists anemia, amenorrhea, constipation, abdominal pain, cold intolerance, hypotension and hypothermia [(1), p. 343]. Also, until the early 1900's, diseases presenting with cachexia were commonplace and sometimes occult, most notable tuberculosis. Therefore, it was a challenge for the attending physicians to determine whether emaciation was caused by reduced food intake or by a physical disorder, reducing diagnostic clarity.

Another special feature of the German health system is that psychosomatic medicine is established as a medical specialty separate from psychiatry. Psychosomatic medicine deals with psychological, social and biological influences on body functions and the development and therapy of somatic diseases (39). The term “psychosomatic” was introduced by Heinroth, whose 1818 textbook was included in our study. Heinroth urged that diseases should be grasped holistically in their somatic and psychological dimensions and special attention should be paid to the patient's biography. Thus, he saw many mental illnesses conditioned by the individual life stories of the affected patients [(40), p. 60]. Psychosomatic medicine has thrived to become an important independent medical specialty in the German-speaking world (41, 42) and has played a dominant role in the treatment of eating disorders. In the past as well as in present day, patients with eating disorders have been treated not only in psychiatric hospitals but also in psychosomatic clinics. This has resulted in a net reduction of involvement of psychiatrists in the diagnosis and treatment of ED, which may explain their reluctance to address this issue.

### Coverage of Disordered Eating and Eating Disorders in Textbooks

The extent to which the authors of textbooks include disordered eating behaviors in their narratives varies widely. Analysis of the texts reveals continuities, discontinuities and differences over the two centuries. At the beginning of the 19th century, authors mentioned disordered eating behavior only sporadically. Reil, the originator of the term “psychiatry,” made important contributions to neuroanatomical research and was anxious to assign the corresponding correlate in the nervous system to certain body functions and symptoms [(43), p. 19–23]. It is therefore traceable that Reil hypothesized increased appetite signified an overactive nervous system in the gastrointestinal tract in his textbook from 1803 [(24), p. 258]. In the period following Reil, German psychiatry was influenced above all by the psychicists (*Psychiker*), who regarded psychiatric illness in primarily moral terms, as a result of disobedient and godless behavior [(43), p. 35f.]. Heinroth is a leading representative of this movement, for example describing the “starvation cure” as a therapeutic procedure to make disobedient patients docile. Patients on a severely restrictive diet would perceive hunger as a punishment for their behavior, notice their need for help and dependence, and consequently end their disobedient behavior [(25), vol. 2, p. 111–24]. On the other hand, the starvation cure had not only an educational function: According to Neumann, it can be seen as a form of asceticism, which would allow the patient to concentrate on the essential. Therefore, the starvation cure was not a pure punishment, but should also encourage the patient's mindfulness. Besides promoting therapies based on strict moral elements and drawing on religious precepts, Heinroth was one of the first psychiatrists to adopt an individual, patient-oriented approach. He interpreted mental disorders as a result of past pathogenic experiences and consequences of behaviors in the patient's life. This led him to advocate consideration of the patient's biography, social circumstances and lifestyle in tailoring therapy (41). He states in his textbook: “Very much depends on detailed knowledge of the individual situation of the ill person. Often this exclusively explains the whole illness (or at least the most relevant factors) and clearly indicates what will benefit or harm them.” [(25), vol. 1, p. 44]. The therapy should be arranged in a manner that “The entire treatment of the ill person is tailored to the specifics of the illness (.). The factors considered include sex, age, constitution, temperament, most importantly the personality, that is the character, cultivation of the mind, inclinations and habits, finally the individual circumstances of the ill person.” [(25), vol. 1, p. 3] Additionally, he made use of activation, fostered the mental powers of his patients and applied other strategies still used in modern cognitive, behavioral and conversational therapy (44). However, despite Heinroth's promulgation of these therapeutic tools in general, there is no evidence as to whether he applied them specifically to address disordered eating behavior.

A more comprehensive study of abnormal eating behavior begins in the mid-19th century, starting with Griesinger and continuing with Neumann, Krafft-Ebing and Kraepelin with the topic increasing in importance. It is noteworthy that all four authors considered reduced food intake as a trigger for psychiatric illness. This pathophysiological approach is not represented in the earlier or later literature, with the exception of Schneider's current textbook [(5), p. 526]. For Griesinger, this approach exemplifies his psychiatric work, he was one of the leading somaticists (*Somatiker*) who aimed for a scientific basis of psychiatry and interpreted psychiatric disorders as arising from somatic conditions [(45), p. 43–9]. Therefore, it is not surprising that Griesinger considered inadequate nutrition as a possible cause of “melancholy” [(26), p. 153]. However, Griesinger was by no means a pure materialist. He also considered psychological aspects in the pathogenesis of mental disorders: “We consider psychological causes to be the most frequent and productive sources of insanity, both as predisposing factors and most importantly as immediate precipitants of the disease.” [(46), p. 169]. Furthermore, Griesinger emphasized that pathogenesis is strongly subjective and that the observed triggers of mental disorders “are most diverse: sometimes it is a suddenly aroused anger, fright or sorrow in response to an insult, financial loss, shame, a sudden death” [(46), p. 169].

Another achievement of Griesinger was the establishment of asylums attached to small towns (“Stadtasyle”): He did not support the prevailing model of custody of psychiatric patients in remotely located madhouses, instead promoting asylums “preferably in the immediate neighborhood of a small town (.), in order to stay in touch with its habitants” [(46), p. 532]. This reflects Griesinger's early appreciation and enforcement of patients' personal autonomy. Kraepelin gives the most extensive account of disordered eating behavior, albeit his four-volume work is also by far the largest of the textbooks included here. Kraepelin is well-known as the founder of empirical, experimental and scientifically founded psychiatry. In particular, he was interested in the detailed study of the course of diseases [(43), p. 106ff.]. Body weight served Kraepelin as an objective parameter to understand the course of a mental disorder and to frame a prognosis, emphasizing his systematic and scientific approach to psychiatry.

By contrast, in the first half of the 20th century a markedly reduced interest in disordered eating is evident in the psychiatric textbooks selected for in this study. This may be explained by a peculiarity of German-speaking psychiatry history. From 1914 to the mid-20th century, reduced food intake in the sense of AN was not seen as a psychiatric illness, but rather as a symptom of hypopituitarism. The Hamburg pathologist Morris Simmonds had published an article in 1914 in which he reported lethal food refusal due to a malfunction of the pituitary gland, known as “Morbus Simmonds” in the German speaking world and panhypopituitarism in English (47). This publication was particularly well-received in Germany, leading to neglect of psychogenic factors in anorexia (48). Most patients with AN were treated by medical disciplines other than psychiatry. At the Charité in Berlin, the famous surgeon Ferdinand Sauerbruch performed transplantation of pituitary glands. In this case, a calf or cattle pituitary gland was transplanted into the greater omentum. A therapeutic effect, ranging from short-lasting weight gain to complete healing was described. Previously, conservative methods such as oral ingestion of pituitary extract and sugar solutions had already been attempted (49). Even during the post-war period, changes in eating behavior made only marginal appearances in psychiatry textbooks and EDs haven't been described as distinct mental disorders in German textbooks at all (19, 34, 35). Not until the mention of “Anorexia mentalis” by Gerd Huber, did eating disorders receive increasing attention in the German literature and recognition as distinct disorders. Some perspectives still seem current from today's perspective, while others can be considered obsolete: The rejection of physical development to womanhood, as emphasized by Huber as a core symptom, is not mentioned in the DSM-5. Instead, disturbed body image as well as the fear of getting fat are included in diagnostic criteria for AN (1). On the other hand, Huber's description that “anorexia mentalis” can turn into an addiction in the course of the disease appears highly topical. It is subject of current research, which investigates possible connections between these two clinical pictures. Godier and Park identified parallels between addictions and AN in a clinical study, including loss of control, functional impairment, self-harming behavior, and occupational and social limitations as intersections of both disease entities (50).

Rennert also emphasizes an “addictive process” in 1987 in patients with AN. It is also striking that he does not list any physical illnesses, such as a disturbed pituitary function, as a possible cause of the disease. Rather, he sees “contact-weak, even defiant, idiosyncratic, ambitious, egoistic or anankastic, hypochondriacal and sensitive characters” as forming the predisposition to AN [(20), p. 457]. Describing a purely psychogenic pathophysiology, Rennert is the first of our authors to suggest psychotherapy as a therapeutic tool. The importance of circumscribed areas of the brain in appetite regulation is discussed again in Schneider's current textbook. It highlights the importance of the hypothalamus as well as orexigenic and anorexigenic hormones. However, cognitive behavior therapy is acknowledged as the most effective treatment [(5), p. 422].

### The Presentation of Food Denial as a Dominant Concern

Food supply has varied markedly over the time period of this study. Between 1800 and the 1950's food was often in short supply. For example, during 1816, the “year without a summer” there was a serious famine in Central Europe (51). Later, at the time of the two world wars, food supply in many parts of Europe was catastrophic and hunger omnipresent. The economic upswing starting in the 1960's has introduced an era of unprecedented abundance of food. Over the same period diseases causing inanition such as tuberculosis and chronic anaemias have reduced markedly in prevalence. The net result has been for psychogenic refusal of food to be more conspicuous and more negatively connoted as pathological, favoring the acceptance of AN into the nosology and AN becomes a distinct diagnosis in German-language psychiatric textbooks [(21), p. 270f, (22), p. 543f.]. This change in emphasis has also resulted in reduced food intake being less commonly attributed as a symptom of other psychiatric disorders.

The more recent rise in obesity has also focused attention on disorders of excessive eating. Historically, it appears that increased food intake was not considered as deviant behavior for a long time. This is supported by the interpretation of increased food intake as a sign of convalescence in psychiatric disorders as described above, but also by the beauty ideals of past times. In the 19th century, for example, excessive body mass was still considered a sign of prosperity, even wealth, and not regarded as a sign of abnormal eating habits or even a mental disorder [(52), p. 34f.]. In our sample, mention of BN as a condition with increased food intake is found only in the current textbooks by Schneider and Tölle/Windgassen (5, 23). Although in 1879, Krafft-Ebing used the term “bulimia” in relation to increased appetite, he did not mention measures to avoid weight gain that are regarded as typical for this disease today (28). In 1974, Huber still equated “bulimia” with hyperphagia. As early as 1972, Otto Doerr-Zegers had aptly described “Bulimia nervosa” as a distinct mental disorder. He and Gerald Russell are considered to be the first to describe this disease (9, 10). As an eating disorder with increased food intake without compensatory behaviors as countermeasures, BED was first listed in DSM-5 (1). Schneider's latest textbook of 2017 is the only textbook in our sample that mentions this disorder. BED was first described by Albert J. Stunkard who published an apt description of this disease in 1959 (53). However, it took a long time for BED to be included in the DSM and considered as a discrete entity. In the selected textbooks of the past 200 years of German-speaking psychiatry, this mental disorder played practically no role at all.

### Disordered Eating in Mental Disorders

In the textbooks in our sample, decreased food intake and weight loss have been regarded as symptoms of depressive disorders, schizophrenia and somatic diseases. DSM-5 mentions all of those conditions as possible differential diagnoses of AN, underlining that alterations in food intake are still today considered as crucial symptoms of these disorders (1). Additionally, further overlaps between depressive disorders, schizophrenia, medical conditions and AN have been found, regarding their symptoms, pathophysiology, genetics and possible treatment strategies.

### Depression

In depressive disorders, severe weight loss can occur. Changes in weight are mentioned as a diagnostic criterion in DSM-5 (1). Furthermore, depression is the most common comorbidity of patients with AN, prevalence of mood disorders ranging from 31 to 89% (54, 55). Comorbid depressive disorder worsens the outcome and prognosis in patients with AN (56).

Besides the frequent coexistence of these disorders, they share some symptoms, notably menstruation, libido and sleep disturbances occur commonly in both disorders. Shared genetic predispositions (57) and fluoxetine as a treatment in common for both disorders have been reported (58).

The feeling of guilt is a typical symptom and a strong indicator of depression (1). Whether reduced food intake is associated with an increased perception of guilt, as described by Griesinger, Krafft-Ebing, Kraepelin and Rennert (20, 26, 28, 29) in depressed patients, is of current scientific interest in ED research. In people with AN, guilt, anguish, sadness, fear and anger have been found to be associated with eating (59). A recent systematic review found that guilt is not consistently linked to AN and BN presentations, but the relationship is unclear due to a lack of data (60).

### Suicidality

Restrictive eating or self-starvation in patients with AN are currently not interpreted as suicidal behavior, as was perceived by the 19th century psychiatrists Heinroth, Neumann, Krafft-Ebing and Kraepelin (25, 27–29). However, AN has the highest mortality rate amongst all psychiatric illnesses. This is explained by the physical consequences of the disorder, but also the high suicide rate (61). Suicide attempts are a major issue in EDs (62), with suicide the second leading cause of death among individuals with AN (63). Therefore, studies have examined possible risk factors for suicidality in patients with EDs in order to prevent suicides in these patient groups. Known risk factors include frequent purge behavior, poor emotional regulation, childhood abuse and psychiatric comorbidities (64).

### Schizophrenia

Reduced food intake has been listed as a symptom of schizophrenia by numerous authors (20, 21, 23, 26–29, 33). As explanations, the authors mainly described delusions of poisoning, gustatory hallucinations and command auditory hallucinations (21, 26–29). From today's perspective, these are still valid reasons for reduced food intake in patients with schizophrenia (65). Furthermore, overlaps between AN and schizophrenia regarding their symptoms, pathophysiology, genetics, and even their therapy have been discussed recently.

In schizophrenia, eating disorders are much more common than in the general population. Disordered eating is up to 5 times more common in patients with schizophrenia (66). On the other hand, diagnosable psychotic episodes are reported in 10–15% of AN patients (67). Additionally, some symptoms of AN resemble those of schizophrenia. These include odd thinking processes, fixed illogical beliefs or deficits in work and social life that can occur in both disorders (68). Due to these reciprocal findings in AN and schizophrenia, several hypotheses have been discussed as possible explanations: psychoses could be a result of starvation, disturbed body image a result of delusions, extreme fasting a counteraction against weight gain from antipsychotics. AN could even be a prodrome of schizophrenia (69). One study took up the last thought: In the study group considered, an ED preceded 10%, in 5% this was AN. The ED occurred 4–8 years before schizophrenia in these cases; women were more frequently affected than men. As a distinguishing feature, gustatory hallucinations were found only in the group with premorbid EDs. The authors discussed the possibility that patients could represent a distinct subtype of schizophrenia (68).

There are also similarities in pathophysiology: Genome-wide association studies (GWAS) in EDs have been used to calculate genetic correlations between different disorders. It is worth mentioning that significant positive genetic correlations were observed between AN and schizophrenia which suggests an overlap in the biological pathophysiology of both disorders (70, 71).

Furthermore, there are overlaps of AN and schizophrenia concerning both psychotherapies and medication. Aripiprazole and especially olanzapine, two atypical antipsychotics, have been trialed successfully as psychopharmacological treatments of AN and might become the first approved psychotropic drugs for AN treatment (72, 73).

### Historical Disorders

Although hysteria, hypochondria and neurasthenia are considered historical disorders, and their concepts have emerged in other mental disorders, we have found overlaps with eating disorders. A specific question is whether these historical disorders could represent an early appearance of eating disorders in the literature.

Hysteria was covered extensively in the textbooks published in the first half of the 20th century. Comparing the historic descriptions of hysteria with the modern view on AN, we found overlaps, especially for the binge-eating/purging type of AN. As noted above, the authors described refusal to eat as a common symptom of hysteria, resulting in amenorrhea and severe weight loss. Vomiting was considered a common symptom of hysteria, and is described as a possible purge strategy in patients with AN in DSM-5 (1, 29, 31, 33, 35).

On the other hand, we did not find the other main DSM-5 diagnostic criteria, fear of weight gain and disturbed perception of body shape in any of the textbooks. It is possible that in the past patients with AN were diagnosed with hysteria. However, due to the absence of some of the main diagnostic criteria for AN in the descriptions of hysteria, we do not regard this disorder as an early description of AN. Our findings support previous research in the history of psychiatry by Vandereycken and van Deth who discussed possible overlaps between hysteria and eating disorders (74). They also found food refusal and vomiting listed as typical symptoms of hysteria. However, they found no literature describing a disturbed body shape or a fear of gaining weight in this context. Further, vomiting in hysteria was not primarily directed at weight loss, rather it was demonstrative, aiming to attract attention. This is in direct contrast to the behavior of patients with eating disorders, who vomit and purge covertly. On these grounds, Vandereycken and van Deth considered hysteria to be a historical precursor of conversion disorder and not EDs. In conversion disorder, a shift of psychic conflicts to somatic symptoms is seen as the critical trigger for its manifestation (74). This pathophysiological explanation was already in use for hysteria: Karl Jaspers wrote that the hysterical vomiting would be a result of “a painful affect that arises during the meal, but is suppressed, then provokes nausea and vomiting, which persists for months as hysterical vomiting” [(30), p. 176]. Hysteria is not viewed as a distinct disorder in the present day, its precepts having been scattered across dissociative or conversion disorder, neurotic disorders or psycho-reactive syndromes, and histrionic personality disorder.

### Gastrointestinal Symptoms and Eating Disorders

The textbook authors described numerous gastrointestinal medical conditions as possible triggers for food refusal, especially in the 19th century. Most common explanations for reduced food intake were hypersensitivity of the gastrointestinal nervous system, abdominal pain and gastrointestinal inflammation (26, 28, 29). To the present day, somatic diseases are listed as important differential diagnoses of AN in DSM 5, requiring careful evaluation (1).

The combination of restricted eating and gastrointestinal symptoms is commonly observed in patients with EDs and often leads to additional pharmacological treatment with antacids, proton-pump inhibitors, antispasmodics, gastroprokinetics, non-absorbable sugar laxative or hyperosmotic laxatives in patients with EDs (75).

As described by the cited authors in their historic textbooks, the enteric nervous system has a significant impact on appetite. Being part of the “gut-brain-axis,” it registers the amount of consumed food to the CNS. The vagus nerve plays a key role, taking part in the regulation of the digestive system, satiety and food intake (76). Abnormal function of the enteric nervous system has been described in patients with AN, including increased sensitivity to gastric expansion and nutrients in the small intestine (77). This may contribute to the manifestations of eating disorders, though etiological implications are unclear and further research is needed.

Krafft-Ebing, Kraepelin and Griesinger discussed inflammatory changes in the gastrointestinal tract as pathophysiological factors resulting from changes in food intake and eating behavior (26, 28, 29). This could be seen as an early scientific hypothesis that inflammation is involved in the pathophysiology of eating disorders. This idea is currently followed by researchers focusing on cytokine changes in EDs (78), discovering immunologically important genes as relevant in AN (79) and exploring the role of the gastrointestinal microbiome in the development of EDs (80).

### EDs and Brain Areas

In the German textbooks of the 19 and 20th century, brain areas with vegetative functions, frontal areas, the diencephalon, and the pituitary gland were seen as decisive for altered food intake and eating behavior by Bumke, Bostroem, Hoff and Huber (21, 32, 33, 35).

Currently, three main neurocircuits are held to be principally involved in food intake, appetite, body weight regulation and the pathophysiology of EDs: These are the self-regulation, hedonic and homeostatic systems.

The self-regulation system embeds eating in the social context, creates individual values and performs self-regulatory control. Its main center lies in the prefrontal cortex (58). It has been debated whether the enhanced control of food intake in patients with AN might be an effect of augmented control in general (81). An fMRI study showed that patients with AN have deviant folding in the prefrontal cortex, persistent even after weight gain and possibly of pathophysiological significance (82). On the other hand, reduced self-regulatory control has been put forward as a neurocognitive feature of BN and BED. In particular, the uncontrolld binge eating that occurs in these disorders suggests reduced control over behavior. Recent findings of an fMRI study underline the possible impact of the self-regulatory system in BN: Reduced thickness in parts of the prefrontal cortex (orbitofrontal cortex, inferior frontal cortex) was associated with more frequent manifestation of BN symptoms in patients (83).

The function of the hedonic system is to elicit the desire to eat and to evoke pleasure during food consumption. Its neurons and synapses are found mainly in the prefrontal cortex, basal ganglia and thalamus (58). In AN, an altered response to reward stimuli is proposed, affecting eating behavior. For patients with AN, food seems to be less rewarding than for the general population. Patients with AN might be able to ignore food-related rewards (84). Alterations in the reward system do not seem to be confined to eating behavior and persist even after convalescence: A fMRI study showed deviant activity in the reward system when patients were confronted with monetary stimuli (85). In BED, on the other hand, patients show an increased reward from food intake and hedonic eating behavior, which could explain the increased food intake (86). For BN, there have been no consistent findings, and studies have found both increased and decreased reward responses to food stimuli (87).

The homeostatic system integrates peripheral signals of food consumption and energy storages and regulates appetite. The hypothalamus and the pituitary gland play a prominent role in this system (58).

The historical assumption that AN could be a consequence of hypofunction of the hypophysis is considered obsolete today. Although there have been observed alterations in hypothalamic-hypophysis-axis in patients with AN, this phenomenon is not considered to have a pathophysiological impact. It is rather considered as an non-specific consequence of starvation in patients with AN (88), a bodily adaptation to decreased food intake and energy deficit, explaining some of the typical somatic complications of AN. Elevated CRH and ACTH levels lead to hypercortisolism, resulting in lowered bone density. Alterations in the pulsatility of LH-secretion contribute to amenorrhea (89).

From today's point of view, the pathophysiological contributions made by Bostroem and Hoff stand out. In the middle of the 20th century, both authors elucidated brain areas that are still considered to play an important role in food regulation. Bostroem identified frontal brain damage as a possible cause of food cravings [(32), p. 23]. As noted above the prefrontal cortex, as a part of frontal cortex, influences food behavior substantially and is considered to play a role in the pathophysiology of EDs. Hoff, on the other hand, considered lesions of the diencephalon as a possible cause of food cravings [(35), p. 199]. Distinct areas of the diencephalon, namely the hypothalamus and the pituitary gland, are still considered to regulate food intake. This emphasizes the early adoption by Hoff and Bumke of the study of circumscribed brain areas as possible causes or loci of disordered eating. This contrasts with the dominant approach in the 20th century to frame the causality of EDs in terms of social, environmental or cultural issues. Today's research seeks to understand both aspects of this equation in relation to EDs, whereby it is postulated that such social factors, together with increased vulnerability due to altered brain functions, could lead to the manifestation of eating disorders (90).

### Limitations

A systematic literature review according to modern standards is currently not possible for a longitudinal study with historical literature: Historical sources are not included in today's literature databases, the quality standards for scientific work common today did not exist in the past. As a result, the selection of literature using PRISMA guidelines or similar criteria was not possible.

Nonetheless, we have made use of the structured and predefined PRISMA methodical approach as outlined in the PRISMA checklist (91) by describing the rationale for the review, providing an explicit statement of questions being addressed, defining eligibility criteria of the psychiatric textbooks as information sources and describing the selection and data extraction process. We also summarized the evidence in a structured way, addressed the limitations and drew comprehensible conclusions that are justified by the applied methods and the obtained results. Overall, we applied the PRISMA approach and its philosophy as far as possible to adhere to contemporary scientific quality standards and to provide evidence-based scientific work. But due to the nature of the historic material, a methodological compromise had to be attained. Whilst his may be seen as a limitation it could also be perceived as the result of successful interdisciplinary cooperation.

Historical textbooks are difficult to compare with each other, especially due to the substantially changed terminology over the course of time. For the selection of literature, we decided on the following strategy: We intentionally chose psychiatric textbooks because we wanted to present the knowledge that was considered valid at the time, representing the most common and generally accepted views of medical doctors and psychiatrists on disordered eating in the past 200 years. The resulting inclusion criteria are noted in the methods section. Articles in journals were not taken into consideration, as they did not necessarily represent valid and widespread knowledge. The main intention was not to compare the sources with each other, but rather to chart how disturbed eating behavior has been viewed over the course of the study period, 1803–2017. This allows a representation of progressions, continuities and breaks over time that would not be possible with a cross-sectional study. A further limitation is the small number of publications considered. This makes it impossible to obtain an all-encompassing overview of all the concepts discussed regarding disordered eating behavior. However, by limiting the literature selection to relevant textbooks from acknowledged authors, it is possible to obtain an overview of the predominant and widespread concepts on disordered eating behavior and eating disorders, gaining an impression how patients suffering from those disorders have been diagnosed and treated in psychiatric clinics.

In this article, we have contrasted the content of important German textbooks with the DSM classification even though we are aware that this comparison may be misleading. It would have been more adequate to compare English language textbooks of psychiatry over the same period with their German counterparts, covering the same breadth of perspectives. Similarly, with the French literature, which also has some historical alignment. Such an approach would be highly informative, but it was well-beyond the scope of the current study. The emphasis on the DSM classification was chosen largely to emphasize two points, firstly the convergence of illness concepts in German with those in the English-speaking world and, secondly, to illustrate how much the illness concepts and disease categories of disordered eating are in flux. This may be seen as supporting the need for a detailed historical analysis to illustrate areas of firm agreement over two centuries vs. areas which have only consolidated in the past decades.

## Conclusion

Although AN was first described in the late 19th century and received broad attention in France and the United Kingdom, it took a long time until AN was recognized as a distinct mental disorder in German-speaking psychiatry. We found the first mention in Huber's textbook of 1974, a full century after its first description in France and England. BN and BED appeared only recently in German textbooks. However, throughout the past two centuries, disordered eating has been recognized as a symptom of various mental disorders, including those classified today as depressive disorders, schizophrenia and bipolar disorder. Interestingly, there are numerous overlaps between those mental disorders and EDs, and DSM-5 features some of them as possible differential diagnoses. Similarities can be found in their genetics, pathophysiology, symptoms and therapy, possibly leading to promising new therapeutic approaches for EDs in the future.

## Data Availability Statement

The datasets generated for this study are available on request to the corresponding author.

## Author Contributions

HS and LB conceptualized the study. HS provided methodological advice for literature review, provided information regarding the history of psychiatry, and the selected psychiatrists. HH provided professional support and made additional remarks regarding the current state of scientific knowledge of eating disorders. KK revised language style of the article and provided professional remarks. LB examined the selected textbooks, undertook a literature search, and wrote the first manuscript draft. All authors contributed and have approved the final manuscript.

## Conflict of Interest

The authors declare that the research was conducted in the absence of any commercial or financial relationships that could be construed as a potential conflict of interest.
